# Supplementary material for: Development of a Proposal for a Program to Promote Positive Mental Health Literacy among Adolescents: A Focus Group Study
Source: Int J Environ Res Public Health. 2023 Mar 10;20(6):4898. doi: 10.3390/ijerph20064898 (PMC10049546; doi:10.3390/ijerph20064898)
Supplement: Supplementary file 1 [file ijerph-20-04898-s001.zip › Suppl Materials_v2/S1_Table with the participants characteristics.pdf]

**Table S1.** Participants' characteristics (n=11)

| | | | *n* | % | Min | Max | Mean (SD) |
|---|---|---|---|---|---|---|---|
| Professionals | Age | | 9 | | 26 | 57 | 41.88 (9.30) |
| | Sex | Female | 8 | 88.9 | | | |
| | | Male | 1 | 11.1 | | | |
| | Academic qualifications | Bachelor's Degree | 1 | 11.1 | | | |
| | | Master's Degree | 6 | 66.7 | | | |
| | | Doctoral Degree | 2 | 22.2 | | | |
| | Professional activity | Child Psychiatrist | 1 | 11.1 | | | |
| | | Psychologist | 1 | 11.1 | | | |
| | | Specialist Nurse in MHPN | 5 | 55.6 | | | |
| | | Researcher | 1 | 11.1 | | | |
| | | Teacher of Basic Education | 1 | 11.1 | | | |
| | Years of professional experience (mean) | | 9 | | 5 | 28 | 18.33 (7.97) |
| | Years of experience with adolescents (mean) | | 9 | | 0 | 27 | 12.22 (8.27) |
| Adolescents | Age | | 2 | | 14 | 14 | 14 (0) |
| | Sex | Female | 1 | 50 | | | |
| | | Male | 1 | 50 | | | |
| | School year | $9^{th}$ grade | 2 | 100 | | | |

Abbreviations: MHPN, mental health and psychiatric nursing; *n*, number of cases; SD, standard deviation.
