# Supplementary material for: Development of a Proposal for a Program to Promote Positive Mental Health Literacy among Adolescents: A Focus Group Study
Source: Int J Environ Res Public Health. 2023 Mar 10;20(6):4898. doi: 10.3390/ijerph20064898 (PMC10049546; doi:10.3390/ijerph20064898)
Supplement: Supplementary file 1 [file ijerph-20-04898-s001.zip › Suppl Materials_v2/S2_Tree Nodes.pdf]

Tree Nodes

Name		Archives	References	Created on	Created by	Modified on	Modified by	
Intervention Program Proposal - Construction		19	285	12/09/2022 16:29	JN	17/01/2023 23:04	JN	
1.0. Structure		18	152	12/09/2022 16:30	JN	17/01/2023 23:04	JN	
1.1. Context		7	9	12/09/2022 16:44	JN	09/01/2023 20:04	JN	
1.2. Format		6	8	12/09/2022 16:44	JN	17/01/2023 12:57	JN	
1.3. Contents		15	54	12/09/2022 16:44	JN	17/01/2023 20:11	JN	
1.4. Length and Frequency		14	30	12/09/2022 16:47	JN	16/01/2023 23:20	JN	
1.5. Pedagogical Methods		9	13	12/09/2022 16:48	JN	17/01/2023 20:11	JN	
1.6. Pedagogical Techniques		10	17	12/09/2022 16:48	JN	17/01/2023 20:11	JN	
1.7. Resources		6	11	12/09/2022 16:49	JN	17/01/2023 20:11	JN	
1.8. Denomination		8	10	12/09/2022 16:49	JN	17/01/2023 00:28	JN	
2.0. Participants		11	35	11/01/2023 20:31	JN	17/01/2023 23:04	JN	
2.1. Target Group		7	18	11/01/2023 20:32	JN	17/01/2023 00:09	JN	
2.2. Program Facilitators		8	17	11/01/2023 20:32	JN	17/01/2023 20:11	JN	
3.0. Assessement		16	45	12/09/2022 16:41	JN	17/01/2023 23:04	JN	
3.1. Timings		14	23	12/09/2022 16:55	JN	17/01/2023 00:09	JN	
3.2. Evaluation Instruments		12	22	12/09/2022 16:55	JN	17/01/2023 20:11	JN	
4.0. Other Components		15	53	12/09/2022 16:42	JN	17/01/2023 23:04	JN	
4.1. Planning, Articulation and Adaptation		9	17	08/01/2023 20:53	JN	17/01/2023 20:11	JN	
4.2. Involvement		7	18	08/01/2023 20:53	JN	17/01/2023 20:14	JN	
4.3. Training		3	4	08/01/2023 20:53	JN	16/01/2023 23:20	JN	
4.4. Special situations		7	11	08/01/2023 20:53	JN	17/01/2023 20:11	JN	
4.5. Partnerships		1	1	08/01/2023 20:54	JN	17/01/2023 20:11	JN	
4.6. Referral		1	2	09/01/2023 18:35	JN	17/01/2023 12:57	JN	

Generated by the software Nvivo 12
